# Two Cases of Pituitary Stalk Interruption Syndrome in Syrian Children

**DOI:** 10.1155/2020/2039649

**Published:** 2020-03-12

**Authors:** Ibrahim Alali, Reem Saad, Younes Kabalan

**Affiliations:** ^1^Al-Assad University Hospital, Damascus University, Damascus, Syria; ^2^Al-Mowassat University Hospital, Damascus University, Damascus, Syria

## Abstract

Pituitary stalk interruption syndrome (PSIS) is an extremely rare cause of growth failure and delayed puberty. It can be diagnosed by magnetic resonance imaging (MRI) of the hypothalamus and pituitary gland, showing an ectopic or absent posterior pituitary, an absent or interrupted pituitary stalk, or small anterior pituitary, in combination with growth hormone or other pituitary hormone deficiencies. The exact etiology of PSIS is unknown. In this article, we describe two cases of PSIS in Syria which are, as far as we know, the first published cases.

## 1. Introduction

Growth failure is one of the most common reasons for referral to the endocrine clinic. Although the most frequent causes of short stature are constitutional and genetic, endocrine disorders, especially growth hormone (GH) deficiency, should always be considered [1]. Pituitary stalk interruption syndrome (PSIS, ORPHA 95496) is an extremely rare disorder, but its exact prevalence is still unknown [2]. This syndrome is a congenital anomaly of the pituitary gland characterized by GH deficiency (with or without other pituitary hormone deficiencies) along with radiological features of a thin or interrupted pituitary stalk, an ectopic or absent posterior pituitary, or a hypoplastic or absent anterior pituitary [3, 4].

In this article, we describe two cases of PSIS that were diagnosed in boys at different ages from Syria.

## 2. Case 1

A 7-year-old boy was referred to the endocrine clinic for short stature. He was born at full term without complications. His birth weight was 3 kg, and his family history was unremarkable. During childhood, he had all his vaccinations and had not been hospitalized.

On examination, he seemed well without any abnormal facial features, and he was 19 kg in weight and 101 cm in height (body mass index (BMI) 18.62 kg/m^2^). Compared to his midparental predicted length (119 cm), he was 3.6 standard deviation (SD) points below expected. Heart and breath sounds were normal and so was the abdominal examination. Testicular volume was 3 ml each, measured by using a Prader orchidometer. Pubic hair distribution and penile size (3 by 1 cm) were both consistent with Tanner stage 1.

**Figure 1:**
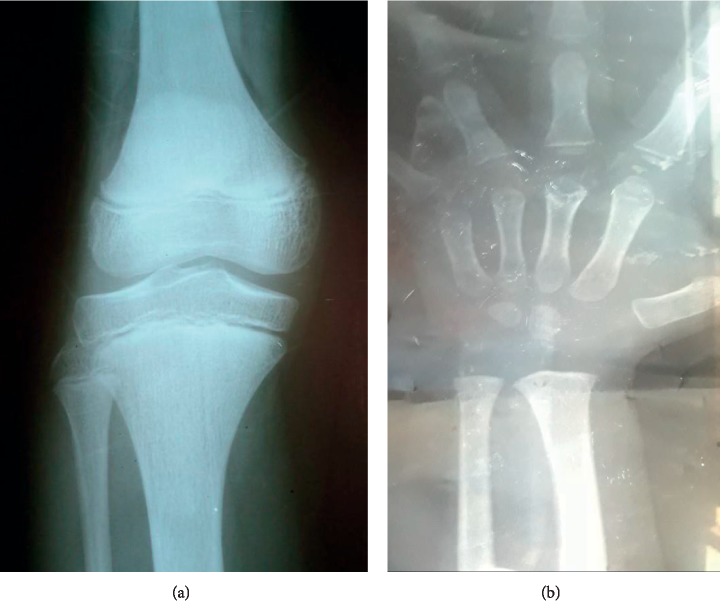
(a) The left hand and wrist bones of the first patient which is consistent with a bone age of 3 years. (b) Posterior-anterior image of the left knee of the second patient reveals the open epiphysis of the growth plate.

**Figure 2:**
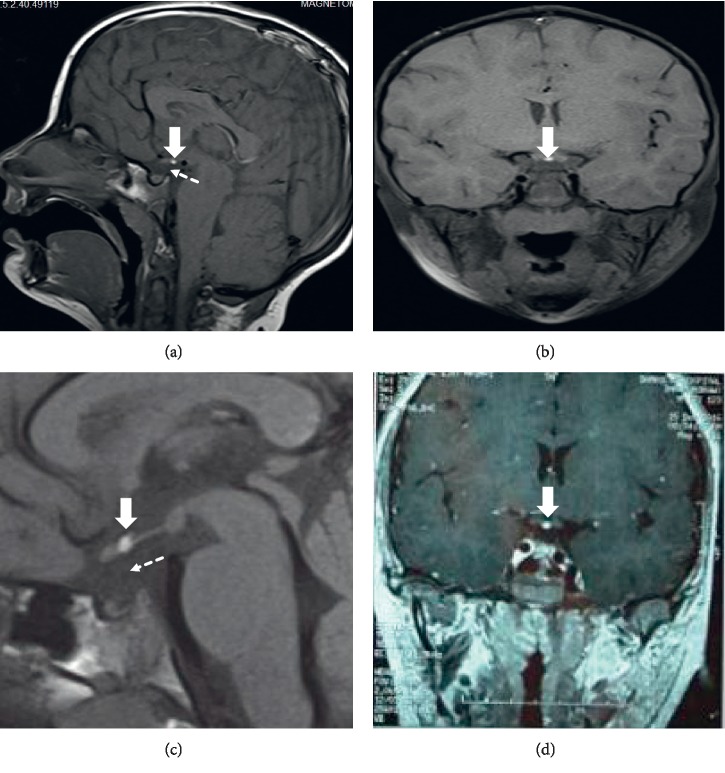
(a) The first patient's sagittal MR image without contrast for the pituitary; the solid arrowhead points to the posterior pituitary lobe (bright spot), while the dashed arrowhead points to the supposed pituitary stalk which is absent. (b) Coronal image without contrast for the first case shows the ectopic bright spot close to the optic chiasm. (c) Noncontrast sagittal view for the pituitary in the second case where the solid arrow points to the ectopic bright spot and the dashed arrowhead points to the absence of pituitary stalk. (d) Contrast coronal image for the second case.

Routine blood panel and urine and stool tests were within normal range, except for mild anemia (Table 1). Plain films of the left wrist were ordered and were compatible with a bone age of a three-year-old (>2 SD below chronological age), as shown in Figure 1. A GH stimulation test conducted with 75 *μ*cg of clonidine was consistent with GH deficiency. Other pituitary hormones were also evaluated and revealed central hypothyroidism and adrenal insufficiency (Table 2). Pituitary MRI was also performed to evaluate for anatomical abnormalities and revealed an ectopic posterior pituitary lobe along with an absent pituitary stalk (Figures 2(a) and 2(b)), confirming the diagnosis of pituitary stalk interruption syndrome (PSIS).

## 3. Case 2

A 17-year-old boy presented to the endocrine clinic for short stature as well as absence of secondary sexual characteristics.

He was born full term in a breech position without complications and had a birth weight of 2.3 kg. No hypoglycemia or other complications were reported during childhood. On examination, he had no abnormal facial features and looked well. He weighed 43 kg and was 150 cm in height (BMI 19.11 kg/m^2^). Compared to his midparental predicted length (169 cm), he was 3 SD points below expected. Auscultation of the heart and lungs did not reveal any abnormalities. Abdominal exam was normal. Testicular volume was 4 ml each, measured by using a Prader orchidometer. Pubic hair was consistent with Tanner stage 1. Penile size was 2 by 1 cm, consistent with micropenis. No gynecomastia was noted.

Routine blood panel and urine and stool tests were normal (Table 1). A plain film X-ray of the left wrist was consistent with a bone age of 13 years. Plain film X-ray of the knee showed open epiphyseal plates (Figure 1). Insulin-like growth factor 1 (IGF1) was low. Gonadal hormone assessment and GH stimulation test, conducted with 150 *μ*cg clonidine after sex hormone priming with 100 mg IM testosterone, showed GH deficiency as well as hypogonadotropic hypogonadism (Table 2). Other pituitary hormones were also evaluated and revealed central hypothyroidism and adrenal insufficiency. Pituitary MRI was performed (Figures 2(c) and 2(d)) and again confirmed the diagnosis of pituitary stalk interruption syndrome (PSIS).

Results of echocardiography, kidney ultrasound, and ophthalmic evaluation were normal for both patients.

## 4. Patients Follow-Up

Our first patient was treated with hydrocortisone 15 mg daily and then levothyroxine 50 *μ*cg daily, followed by recombinant GH 1.7 IU SC daily. Within three months, he became euthyroid and gained 3 kg weight and 3 cm height.

Our second patient was treated with hydrocortisone 15 mg daily, levothyroxine 75 mcg daily, and testosterone 100 mg monthly. He was later referred to a specialized center for recombinant GH replacement.

## 5. Discussion

Pituitary stalk interruption syndrome (PSIS) was initially described and defined by Fujisawa et al. in 1987 [3]. PSIS is diagnosed primarily based on the radiologic findings mentioned above along with permanent GH deficiency and one or more other pituitary hormone deficiencies [2, 3].

The exact prevalence of this syndrome remains unknown. Less than 1000 cases were reported in the literature until 2010 [2]. Its prevalence as a cause of GH deficiency is estimated to be around 4% [5]. The age at diagnosis differs according to the severity of the hormone deficiency. When PSIS presents at birth, hypoglycemia and failure to thrive are the most common symptoms. In childhood, growth retardation is usually the presenting complaint, whereas delayed puberty is usually the chief complaint when it manifests in adolescence and early adulthood [4, 6]. Although these two patients had multiple hormone deficiencies, diagnosis was established relatively later in life.

Breech delivery, cesarean section, and neonatal hypoxemia were suggested as potential causes of this syndrome [7]. However, many case series found an antenatal origin [4, 8, 9]. Genetic mutations were also detected in familial cases, such as HESX1, LHX4, SOX3, PROKR2, and OTX2 genes. Of note, genetic mutations are thought to contribute to less than 5% of PSIS cases [4, 8]. Zwaveling-Soonawala et al. found additional suggested genes for isolated PSIS (DCHS1, ROBO2, CCDC88C, and KIF14) and one for syndromic PSIS (KAT6A). Presence of many probable pathogenic genes and heritage from normal parents are implicative of polygenic etiology of sporadic cases of PSIS [10]. Similar findings were found in Guo et al.'s study in which a group of gene mutations was identified in 92% of the patients and genes NCOR2, ZIC2, and NKD2 were found to be mostly associated with Notch, Shh, and Wnt signaling pathways, respectively [11].

While our second patient had a breech delivery, our first patient underwent uncomplicated vaginal delivery and had a negative family history. The presence of multiple hormone deficiency suggests a genetic etiology. However, due to lack of availability of genetic testing assays in our country, this was not performed.

In most published series [3, 4, 8–13], PSIS exhibited a male predominance, similar to what is observed in our cases. The underlying reason behind this phenomenon is unknown, though there is a selection bias in such patients since boys, more than girls, are brought by parents for growth assessment.

TSH deficiency was the most common deficit (79.5%) alongside GH deficiency in PSIS patients, and ACTH deficiency also was common (67.5%), followed by FSH/LH deficiency in 65.1% of patients [4].

Our two cases had GH, TSH, and ACTH deficiency. LH/FSH deficiency may be detected in minipuberty or discovered later in peripuberty. Therefore, gonadal assessment should be conducted regularly in patients with PSIS [14]. While hypogonadism was detected only in the second case, gonadal axis was not assessed in the first patient given his young age.

The incidence of extrapituitary manifestations in PSIS is high [8]. Midline defects affect mainly the brain and eyes. Extracerebral abnormalities also include defects of the heart, skin, and extremities, which highlights the importance of regular examinations and cardiac, ophthalmologic, and cerebral screening [5].

The lifelong prognosis depends on the time of diagnosis and early treatment of hormonal deficiency [8].

Close monitoring of a child's growth over time helps in early detection of growth failure and early treatment of the underlying causes. Rare etiologies such as PSIS should be kept in mind especially in multiple hormonal deficiencies, as it carries good long-term prognosis if diagnosed early on and treated adequately.

Finally, these two cases are, as far as we know, the first published cases from Syria.

## Figures and Tables

**Table 1:** Results of the complete blood count and basic metabolic panel for both patients.

Test	Normal value	First case	Second case
WBC (per mm^3^)	4.5–10.5 × 10^3^	5.2	7.2
Hb (g/dL)	11.5–13.5	10.2	13
PLT (per mm^3^)	150–450 × 10^3^	343	225
Creatinine (mg/dL)	0.6–1.3	0.7	0.7
Glucose (mg/dL)	60–99	71	95
ALP (U/L)	Up to 936	153	759
ALT (U/L)	Up to 41	32	—
Anti-tTG IgA (*μ*U/mL)	<10	5.3	0.2
Total IgA (mg/dL)	61–348	—	99
Calcium (mg/dL)	8–10.5	8.3	10.4
Phosphorus (mg/dL)	2.5–5.3	4.8	5.3
Urine analysis	—	Normal	Normal
Stool analysis	—	Normal	Normal

WBC, white blood cells; Hb, hemoglobin; PLT, platelets; ALP, alkaline phosphatase; ALT, alanine aminotransferase; tTG, tissue transglutaminase; IgA, immunoglobulin type A.

**Table 2:** Results of hormonal evaluation for both cases along with reference range.

Hormone	Normal value	First case	Second case
TSH (*μ*U/mL)	0.3–4.8	3.8	3.5
Free T4 (ng/dL)	0.8–1.8	0.557	0.668
IGF1 (ng/mL)	247–481	—	64.1
Basal GH (ng/mL)	—	0.506	<0.05
GH after 30 m	Any test more than 10	0.513	<0.05
GH after 60 m		0.540	<0.05
GH after 90 m		0.723	<0.05
GH after 120 m		0.719	<0.05
Testosterone (ng/mL)	2–9	—	1.98
LH (mU/L)	2–12	—	1.43
FSH (mU/L)	1.5–12.4	—	<1
8 AM cortisol (*μ*g/dL)	5–25	3.74	3.9
ACTH (pg/mL)	7.2–63	—	24.52
Prolactin (ng/mL)	2–18	—	18.9

TSH, thyroid-stimulating hormone; LH, luteinizing hormone; FSH, follicle-stimulating hormone; ACTH, adrenocorticotrophic hormone
